# The role of a deployed British Army dental team on exercise Immediate Response 24

**DOI:** 10.1038/s41415-026-9880-7

**Published:** 2026-08-28

**Authors:** Sunmeet Kandhari, Christopher Harper, Tim Davies

**Affiliations:** 771377672019045361522 Armoured Medical Regiment, Tidworth, England, United Kingdom; 590615911121422566709 Armoured Medical Regiment, British Army Land Forces Headquarters, Andover, England, United Kingdom

## Abstract

Dental morbidity remains a significant contributor to disease non-battle injuries during military operations, posing a threat to operational effectiveness by causing debilitating pain, necessitating evacuations, and diverting limited medical assets.

This paper explores the role and integration of a forward dental team during Exercise Immediate Response 24, with a particular focus on the use of a repurposed dental support vehicle (DSV). With the DSV, the dental team were able to deliver emergency, routine and preventive dental care closer to the frontline, reducing the need for evacuations and minimising downtime for affected personnel. This demonstrated how mobile, rapidly deployable dental teams could maintain the oral health of a deployed population at risk and prevent dental emergencies from escalating.

While designed for military operations, the concept offers insights for civilian dental teams, particularly those practising within remote or austere locations where conventional dental infrastructure is limited. The lessons learned from the exercise, especially around power supply, mobility, and team versatility, are directly translatable to civilian practice making this a valuable case study for dentists interested in global health, emergency response, or mobile dentistry.

## Key points

- Dental morbidity is a significant contributor to disease non-battle injuries, potentially impairing combat effectiveness.
- Deployable British Army dental teams are critical for maintaining fighting power, preventing dental emergencies and reducing the need for evacuations away from the frontline.
- Lessons from Exercise Immediate Response 24, such as rapid deployment, mobility, digital integration, and team versatility, highlight the potential of forward dental care in both military and civilian contexts.

## Introduction

Modern military operations are increasingly characterised by rapid manoeuvre, dispersed personnel, deep precision strikes and drone warfare.^1^ In this context, every element of a military force must be able to operate independently in order to maintain operational effectiveness.

Dental morbidity, often a significant contributor to disease non-battle injuries (DNBI)^2^^,^^3^^,^^4^^,^^5^ has the potential to impair combat readiness by causing pain, necessitating evacuation and diversion of valuable medical assets. As the British Army continuously refines its tactics and doctrine, the forward dental team concept has re-emerged as a tactical solution to provide dental care closer to the front line.

**Fig. 1:**
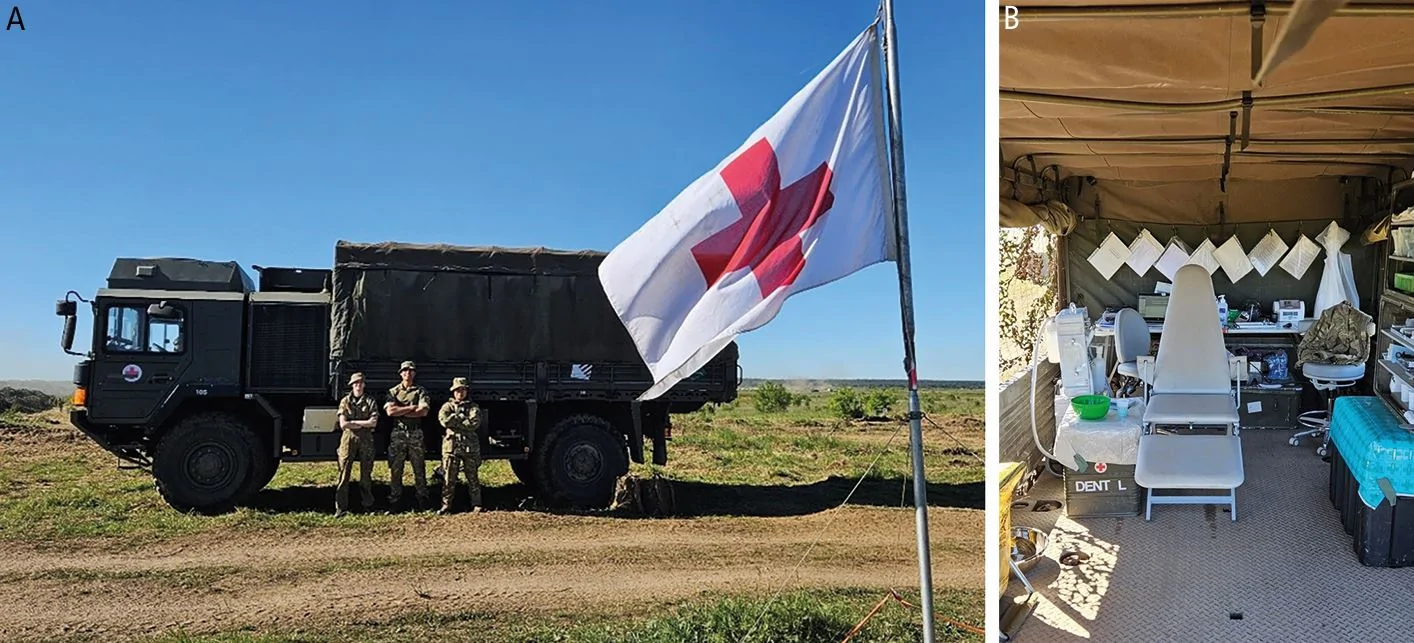
The dental support vehicle

This article examines the deployed dental team's ‘forward' role on Exercise Immediate Response^6^ (Ex IR), exploring its concept, composition and integration into an overarching medical plan with a summary of the lessons learnt from Ex IR. In doing so, it also highlights the potential benefits and constraints of placing dental capability ‘forward' in modern military environments as attempted by the Ex IR dental team through the use of a repurposed truck called the dental support vehicle (DSV) (Fig. 1). Furthermore, the analysis explores how the lessons learnt from Ex IR may be applicable to civilian dental teams, particularly in remote, humanitarian, or disaster responses.

### Background

Historically, the provision of deployed healthcare in the British Army has always evolved in response to changing operational environments. Military exercises, such as Saif Sareea 3^7^ demonstrated that small pre-hospital treatment teams, deployed close to the frontline, can reduce the time from injury to definitive treatment. By enabling this rapid intervention and return of personnel to their duties, these forward-positioned teams serve as force multipliers, maintaining unit strength and operational tempo. This principle of bringing medical care to the point of need equally applies for dental support.

Having long been recognised as one of the top five causes of disease non-battle injuries,^2^^,^^3^^,^^5^ dental morbidity can have a significant effect on the operational effectiveness of the fighting force. These issues, ranging from toothache to blast injury trauma, can lead to evacuations that compromise unit cohesion and mission success. In well prepared forces, dental care^8^ is often provided to ensure service personnel are dentally fit for operations, thereby reducing the likelihood of dental emergencies once deployed. However, despite these efforts there remains a significant burden of oral disease experienced which cannot be fully eliminated by preventative care. As such, forward dental teams can serve as potent force multipliers^9^ by providing both emergency and routine care closer to the frontline. Within this, the operational environment poses inherent challenges^10^ that require forward dental teams to remain adaptable when compared to routine provision in the firm base:

- Austere conditions – remote or frontline locations often have minimal infrastructure, lack basic amenities, or offer limited protection from environmental extremes
- Limited equipment – as space and transport are at a premium, teams may only be able to work with essentials and may lack access to more sophisticated equipment or laboratory support
- Hostile threats– the risk of enemy action, indirect fire, or other dangers may require immediate evacuation or disrupt the ability to provide timely medical support.

These examples illustrate why operational military dental care can fundamentally differ from routine civilian practice. For instance, a severe toothache that may be resolved with a timely trip to a local dentist could instead incapacitate a soldier for several days in an environment where evacuation may be impossible due to weather, enemy activity, or a demanding operational tempo. While these challenges are characteristic of military operations, civilian dental teams operating in disaster zones, rural communities, humanitarian missions, or conflict-affected areas often face analogous constraints as they may similarly feature damaged or non-existent infrastructure, finite resources, and security or logistical disruptions that impact care delivery.

Given these operational realities, it is essential that dental care be available at or near the point of need to maintain operational readiness of the fighting force. The concept of a forward dental team extends these principles to the frontline, and by embedding dental capability into a mobile, agile platform, a dental team can provide timely intervention that prevents the escalation of dental issues into emergencies that might require evacuation.

**Fig. 2:**
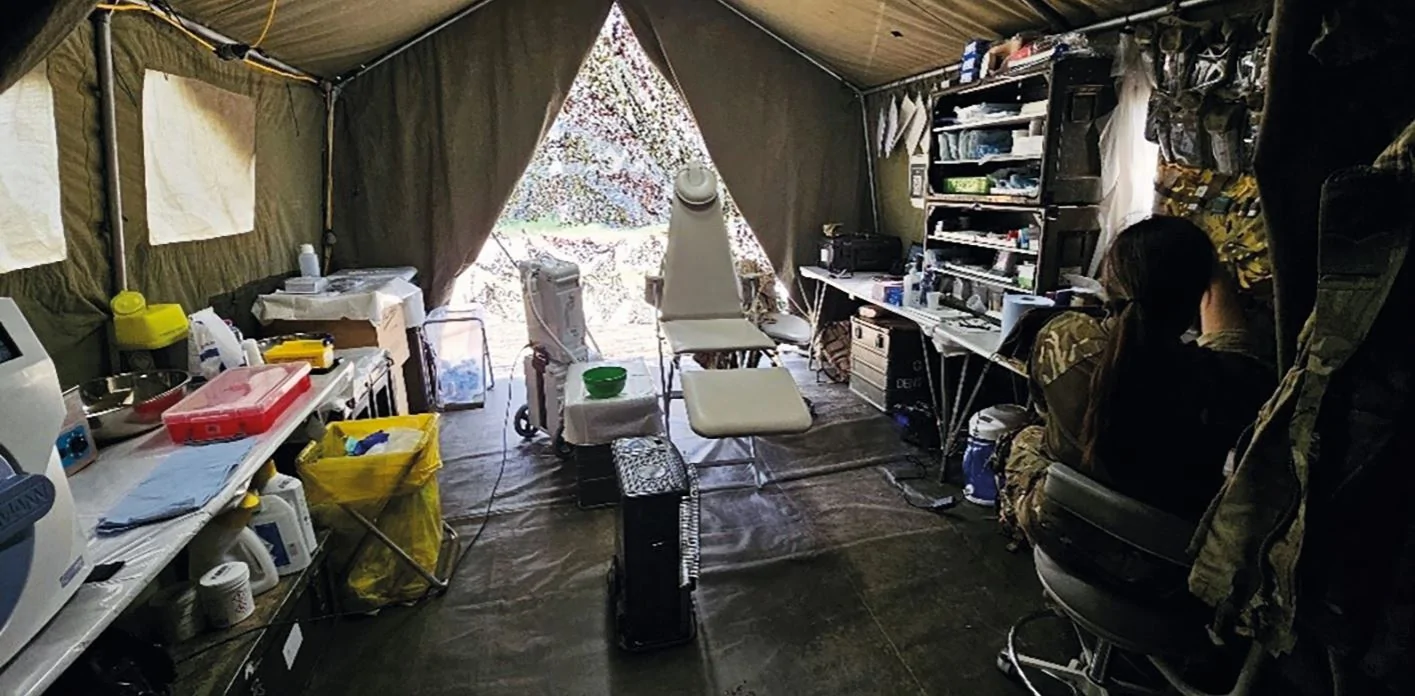
Inside a medical reception station

**Fig. 3:**
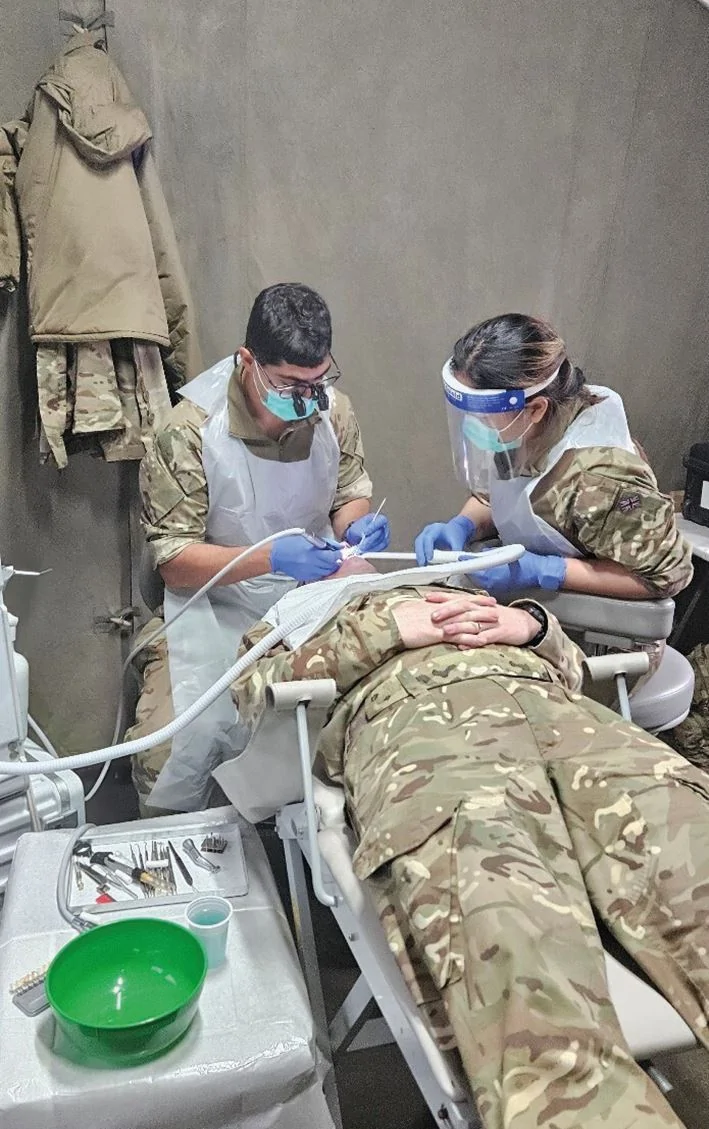
Deployed dental treatment inside an MRS

**Fig. 4:**
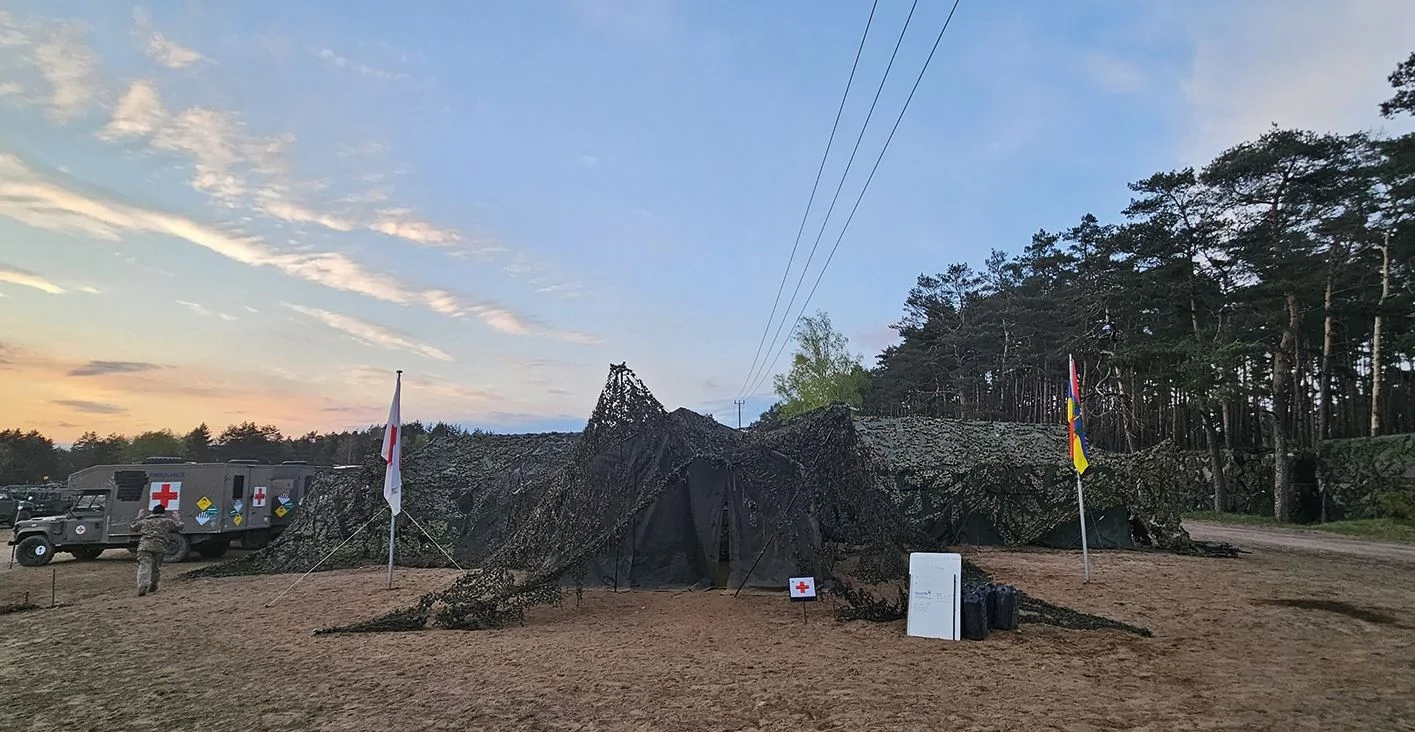
Medical reception station

Ex IR^6^ provided an opportunity to test this concept, particularly through the use of a DSV (Fig. 1), which enabled the forward dental team to operate with greater mobility and a smaller, less conspicuous footprint compared to a more static and less manoeuvrable facility such as a medical reception station (MRS) (Figs. 2, 3 and 4).

While mobile dentistry^11^^,^^12^ is already well established in civilian contexts, the unique demands of military operations, particularly the integration of clinical care with tactical mobility, force protection, and austere environments, offer distinct lessons. By examining how forward dental teams can maintain clinical effectiveness while adapting to operational imperatives, this paper provides insights that may inform both military doctrine and civilian practitioners working in challenging environments or those planning to deploy mobile dental capabilities in future humanitarian, disaster response, or underserved areas.

## The concept of a forward dental team

Principally, a forward dental team is a small, highly manoeuvrable asset, capable of delivering peripatetic dental care to a limited population at risk.^10^ The team is designed to be agile, able to deploy and set up quickly, and operate independently or as an adjunct to a larger MRS (Figs. 2, 3 and 4). Its primary aim is to provide emergency, routine, and preventive dental care closer to the frontline, thereby mitigating the need for medical evacuations and reducing downtime for affected personnel.^9^

One of the key innovations trialled on Ex IR was the integration of the forward dental team with a DSV. This vehicle is a mobile, self-contained dental unit equipped with the necessary instruments and consumables to perform a range of dental procedures (Fig. 1). Traditionally, deployed dental teams have been co-located with an MRS (Fig. 4). However, by using a DSV the Ex IR forward dental team were able to create freedoms away from the MRS and operate independently, achieving full operating capability within 30 minutes of arriving in location. This rapid deployment was made possible by utilising existing deployed dental equipment in the DSV, thereby significantly reducing the time to treatment and the need for complex, static set-ups in forward locations that were closer to the frontline.

The DSV approach provides several practical advantages by allowing the forward dental team to operate from a vehicle, resulting in a smaller and less conspicuous footprint compared to a traditional, more stationary facility such as the MRS. This reduced visibility and increased mobility is crucial in modern conflicts, where forces are constantly under threat from precision strikes and drone warfare.^1^ Additionally, the integration of digital communication systems with the forward dental team further enhances their operational effectiveness. Using Wi-Fi dongles and Android Team Awareness Kits – specialised smartphone applications that enable real-time location sharing, messaging, and coordination on digital maps – the team were able to maintain continuous contact with command centres. This connectivity also enabled remote access to specialist advice, even when internet connections were weak or unavailable, ultimately ensuring that clinical decision making remained aligned with broader military priorities and the activities of the exercise.

Indeed, the mobile dental concept is not new and several civilian healthcare models have demonstrated the ability to overcome geographical barriers in order to provide dental care in remote areas.^11^^,^^12^ However, the military's DSV approach introduces several distinctive elements that could benefit these existing programs. The emphasis on rapid deployment and ability to relocate quickly in response to changing conditions offers lessons for civilian disaster response and emergency healthcare delivery, where traditional mobile units may lack the necessary agility. Additionally, the military's integration of real-time digital communication systems, including mapping applications and secure messaging platforms that function even with limited internet connectivity, represents an advancement over some civilian mobile units that may struggle when operating in areas with limited cellular coverage. Likewise, the ability to maintain continuous contact with specialist consultants and coordinate with central command – what the military calls maintaining ‘situational awareness' – could enhance decision-making for civilian practitioners working in isolation.

Conversely, civilian mobile dental services offer several valuable lessons for military operations. The extensive experience of services like the Australian Royal Flying Doctor Service^13^ in delivering comprehensive dental care over decades, their established protocols for community engagement, integration of wearable technology and artificial intelligence assisted diagnostics could inform military planning for sustained operations. Equally the focus on culturally appropriate care delivery, particularly in Indigenous communities, provides important insights for military teams operating in diverse cultural contexts during peacekeeping or humanitarian missions.

Ultimately, mobile dental units, whether military or civilian, improve health outcomes for underserved populations by delivering care where people live rather than requiring them to travel long distances. This point of need approach reduces barriers to access and alleviates pressure on centralised healthcare facilities, benefits that are equally relevant to isolated communities, disaster zones, and military field operations.

## Composition of a forward dental team

Typically, a forward dental team is deliberately small to maximise its mobility and agility.^10^ The core composition usually includes one dental officer, acting as the clinical lead, and one or two dental nurses (DNs) with additional capability as drivers. This lean structure is designed to ensure that the team is self-sufficient, with the DNs providing both chair-side assistance and the enhanced skills to operate the vehicle when required.

Outside of a vehicular platform, essential equipment includes a portable dental unit, intra-oral radiography, sterilisation equipment, and dependant on the operational context and anticipated demand consumables necessary to treat between 30 and 300 patients before resupply. When deploying forward, the amount of kit carried is carefully tailored to balance the expected patient load with constraints such as space, weight, and the tempo of operations. The portability of these components meant that the forward dental team could be quickly repositioned within the operational area as needed. However, it should be noted that the team is reliant on other military units in the area for security and logistical support, an inherent trade-off when deploying small, dispersed units in high-threat environments.

Furthermore, training plays a crucial role in ensuring the forward dental team's effectiveness. Military dental personnel must maintain proficiency across a broad spectrum of competencies not typically required in civilian practice. Beyond upholding the same rigorous clinical standards expected of any dental professional, they must be proficient in basic soldiering skills such as fieldcraft, weapons handling and navigation, as well as understand the nuances of delivering effective clinical care with limited equipment in austere environments. Crucially, military dental teams develop proficiency across overlapping competencies, with team members gaining a comprehensive understanding of each other's roles and responsibilities. This creates workforce resilience that allows critical services to continue even when one member becomes incapacitated or must attend to other duties.

This military model of cross-training offers valuable insights for civilian practice, particularly in resource-limited or emergency settings. While civilian dental practice may not require the same tactical skills as military operations, the principle of cross-training team members to enhance versatility and resilience could prove equally valuable in underserved areas, disaster response scenarios, or any setting where dental teams must operate with limited resources or personnel. By ensuring that personnel can adapt flexibly to changing circumstances and maintain continuity of care when team members are unavailable, this approach demonstrates how strategic workforce development can strengthen a team's ability to deliver consistent, high-quality care despite operational constraints.

## Role within the overall medical plan

The forward dental team is an integral component of the overall deployed medical plan, particularly within the framework of force health protection. Its role is multifaceted, encompassing not only the immediate provision of dental care. It also contributes to broader operational objectives such as triage, casualty management, and prevention of dental emergencies that could lead to more severe complications

In Ex IR, by operating forward of the MRS, the forward dental team were able to ensure that dental emergencies such as pericoronitis and toothache were managed at the point of need. This rapid response capability is vital in a combat environment, where the consequences of dental pain or infection can extend far beyond individual discomfort, affecting unit cohesion, morale, and overall mission readiness.

In practical terms, the forward dental team's contributions can be summarised as follows:

- Provision of emergency dental treatment to mitigate acute pain and prevent the escalation of dental issues
- Delivery of routine and preventive care to maintain the overall oral health of the deployed force, thereby reducing the risk of dental emergencies
- Rapid deployment and setup that allows dental care to be provided without necessitating lengthy evacuation routes or the establishment of static facilities
- Integration with broader medical command and control structures ensuring that dental care is seamlessly incorporated into the overall operational picture.

In essence, the forward dental team bridges the gap between the traditional firm base dental services and the dynamic needs of a forward-deployed force. Its ability to provide immediate, mobile dental care not only improves individual health outcomes but also contributes significantly to the operational sustainability of the force.

## Lessons learnt from Exercise Immediate Response 24

Ex IR provided an invaluable opportunity to trial the forward dental team concept and assess the operational viability of the DSV in a simulated high-threat environment. Several key lessons emerged from this exercise:

- Rapid deployment and setup: one of the most impressive achievements was the team's ability to become fully operational within 30 minutes of arrival at a new location. This rapid setup is crucial in remote areas where delays in providing care can significantly impact community health outcomes. The trial demonstrated that with proper planning and team training, a mobile dental unit can be deployed quickly to wherever it's needed most. This capability proves equally valuable for emergency dental response after natural disasters or rural outreach programs, where establishing temporary clinics in existing buildings might take considerably longer
- Flexibility and accessibility: using a vehicle-based platform provided clear practical advantages. The mobile dental team could relocate easily as needs of the population changed, serving multiple sites without requiring permanent infrastructure. This mobility enhances the team's ability to respond to urgent dental needs whilst keeping the physical footprint small, making it easier to set up in areas with limited space. While the original context involved security considerations, civilian teams benefit from this flexibility by serving multiple remote locations more efficiently. Disaster response teams particularly benefit from the compact design, as they can navigate damaged roads and infrastructure more easily, reaching isolated populations that large static facilities might struggle to access
- Communication and integration: the trial highlighted the importance of reliable communication systems. By using portable Wi-Fi devices and communication tools, the mobile dental team were able to maintain connectivity with command centres and the broader healthcare network. This digital integration enabled quick decision making, allowed for remote consultation when necessary, and ensured the dental team could access real-time support even in areas with limited internet connectivity. However, if these are to be used in a military context, the practicalities of this require further investigation as the reliance on digital infrastructure may result in a larger electronic signature, which in turn may identify any dispersed elements to a hostile adversary^14^
- Logistical and power supply challenges: despite many successes, the exercise revealed that power supply and logistics remain fundamental considerations for any mobile deployment, whether military or civilian. Chief among these is the issue of power supply. Here, the forward dental team required a bespoke power source known as a lightweight field generator as well as prior logistical planning to request additional equipment in order to operate sterilisation units and other power-dependent devices over a prolonged period. Without a reliable and portable power solution, the teams' capacity to treat patients is limited by the frequency at which instruments can be sterilised. Looking forward, work is underway to explore battery powered alternatives for quieter, more flexible operation; however, the military could benefit significantly from studying and adapting established civilian best practices rather than developing entirely new systems
- Training and versatility: the exercise emphasised the need for comprehensive training to ensure every team member can work effectively in challenging environments with limited resources. Cross-training is also important, and DNs should be able to fulfil multiple roles including vehicle operation and clinical support, enabling teams to function independently with minimal staffing.

## Conclusion

Through preventing dental emergencies from escalating, forward dental teams play a critical role in maintaining fighting power and operational effectiveness. The forward dental team concept trialled during Ex IR demonstrated that high quality dental care can be delivered rapidly and effectively at the edge of the battlefield. By projecting dental capability forward via the DSV, the team reduced the need for costly and time consuming evacuations^15^ while ensuring that dental morbidity did not compromise the health, morale, or combat effectiveness of the force.

The lessons learnt from Ex IR, rapid deployment, enhanced mobility, effective digital integration, and team versatility, highlight the potential of forward-deployed dental care to adapt to the demands of modern military operations. As the British Army continues to adapt to evolving threats, forward-deployed dental care will undoubtedly play a pivotal role in maintaining fighting power forward. Simultaneously, the lessons from Ex IR provide a roadmap for refining the DSV concept and demonstrate its broader applicability to civilian contexts, ultimately enhancing global health resilience in both military and civilian domains.

## Data Availability

The data supporting the findings of this study are available within the article and its referenced sources. Due to the operational and security nature of the exercise, detailed raw data and internal Ministry of Defence datasets (such as the Dental Intelligence Dashboard) are not publicly accessible. Requests for further information or data access can be directed to the corresponding author, SK, subject to MOD approval and applicable restrictions.
